# Characterization of a rare mosaic X-ring chromosome in a patient with Turner syndrome

**DOI:** 10.1186/s13039-022-00593-2

**Published:** 2022-03-31

**Authors:** Hunjin Luo, Liu Ni, Yi-Qiong Yang, Xiao-Min Zhang, Hongping Huang, Sainan Tan, Chen Ling, Li Liang, Ling Wang, Tang Dan, Shu-Xiang Zhou, Chunliu Yang

**Affiliations:** Women and Children Healthcare Hospital of Zhuzhou, No. 128 Che Zhan Road, Zhuzhou, 412000 Hunan Province China

**Keywords:** Turner syndrome, Ring X chromosome, Fluorescence in situ hybridization, Chromosome microarray analysis

## Abstract

**Background:**

Ring chromosomes can be formed by terminal breaks of two arms of a chromosome and their rejoining, leading to a loss of genetic material. They may also be formed by telomere-telomere fusions with no deletion, resulting in the formation of a complete ring. Mosaic X-ring chromosomes are extremely rare and have highly variable phenotypes. Here, we report a case with a mosaic X-ring chromosome in a patient with Turner syndrome, and we illustrate the unreported complicated mechanism using chromosome analysis and fluorescence in situ hybridization **(**FISH).

**Case presentation:**

A 10-year-old girl of short stature presenting Turner syndrome was admitted to our hospital. The patient’s clinical characteristics were subsequently documented. Genetic analysis showed a karyotype of mostly 45,X[140]/46,X,r(X)[60]. The X ring chromosome was cytogenetically characterized as 45,X/46,X,r(X)(p22.32q21.1), with a length of approximately 74 Mb.

**Conclusions:**

Taken together, we report a rare case with a mosaic X ring chromosome in Turner syndrome and we believe this case expands our collective knowledge of mosaic structural chromosomal disorders and provides new insight into clinical management and genetic counseling for Turner syndrome.

## Background

Turner syndrome (TS) occurs in approximately 1/300 to 1/2500 of newborn girls [1]. Its main clinical presentations are short stature, ovarian hypoplasia, webbed neck, valgus, and low hairline [2–4]. TS is a chromosomal disease derived from a complete or partial deletion or structural abnormality of the X chromosome. In addition, some TS patients carry a small supernumerary marker chromosome (sSMC) whose origin, characteristics, and structure have not yet been identified using traditional chromosome banding technology. In TS, the sSMC usually presents in the form of chimerism, which has its own formation and origin characteristics [5].

TS with an X-ring chromosome-related phenotype can present with mental disorders, learning difficulties, autism spectrum disorders, craniofacial abnormalities, cardiovascular diseases, and skeletal issues, as well as the other presentations associated with Turner syndrome. In addition to the common characteristics of TS, the clinical presentation of these patients depends on the origin, size, gene replication time, genes affected by changes in replication number, degree of mosaicism, and X inactivation status of the sSMC [4]. In the present study, we report a rare case who had common clinical features of TS and annular X chromosome with mosaics and a breaking point confined to the region Xp22.32-q21.1 in the X chromosome.

## Case presentation

A 10-year-old female was admitted to our hospital with a past medical history of short stature and moderate intellectual development. She was born to a 22-year-old G1P1 female at 40 weeks via vaginal delivery, with no special conditions during pregnancy. The patient’s birth weight was 2945 g and the patient’s length was 46 cm. The patient’s family history was unremarkable for immediate family members in terms of Turner syndrome. No other family members presented learning or developmental disabilities and short stature. The patient’s parents denied consanguinity. The proband was developmentally delayed. She was followed by developmental pediatrics and received physical therapy. The clinical features of these cases are summarized in Table 1.

**Table 1:** Clinical features of the case

Clinical detail	case
Age (in years)	10
Height (in cm)	120
Weight (in kg)	23.8
BMI	16.5
Growth retardation	+
Menstrual status	−
Mental retardation	−
Lymphedema	−
Web neck	−
Low set ears	−
Cubitus vulgus	−
Short fourth metacarpals	−
Cardiovascular abnormality	−
Autoimmune disorder	−
High arched palate	−
TSH (μIU/ml)	4.35
FSH (mIU/ml)	82.9
LH (mIU/ml)	17.86
E2	8.06
Development of secondary sexual characters	Breast development—Tanner stage 2,axillary hairs and pubic hairs absent and shield chest
Renal malformation	−
Ultrasonographic report	Ovarian dysplasia

## Methods

A cytogenetic microarray experiment was performed using a cyto-HD array according to the manufacturers’ instructions (Affymetrix, Inc., Santa Clara, CA, USA). Data was analyzed using Chromosome Analysis Suite (ChAS) software (Affymetrix, Inc., Santa Clara, CA, USA). High-resolution chromosome analysis was performed according to standard protocols. Fluorescence in situ hybridization (FISH) was performed using a Spectrum Green-labeled probe for the pericentromeric region of chromosome X(SRY/DXZ1). A total of 25 metaphase and 200 interphase cells were visualized using a fluorescence microscope (Olympus BX53, Japan). Follicular-stimulating hormone (FSH) and luteinizing hormone (LH) were measured using a chemiluminescence assay according to the manufacturer’s instructions (Beckman AU680 Controller Area Network CAN, USA).

## Results

**Fig. 1:**
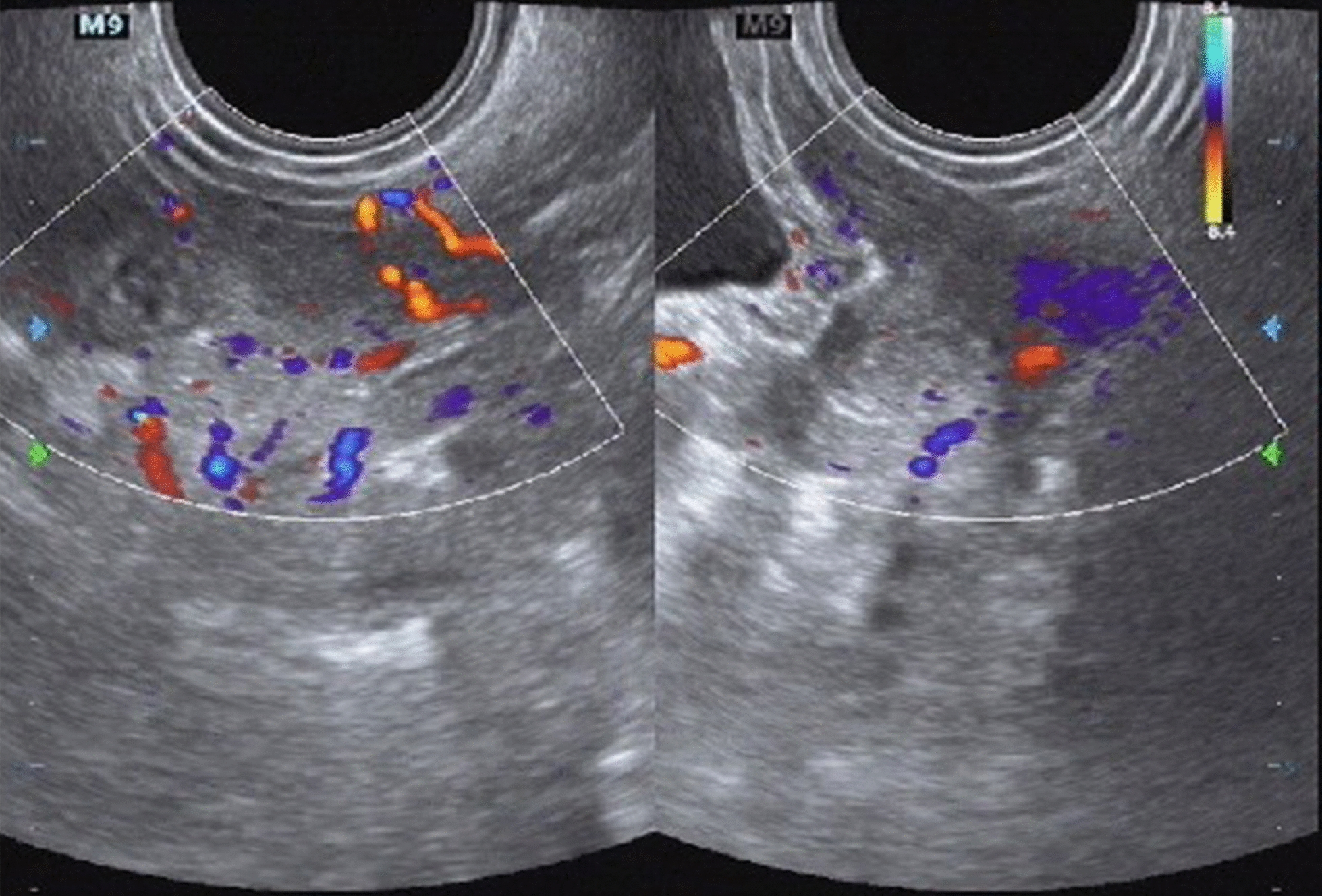
Transrectal ultrasound showing ovarian dysplasia

The case was a 10-year-old girl who was diagnosed with TS in 2021. She had short stature and ovarian dysplasia was observed by ultrasound examination (Fig. 1).

**Fig. 2:**
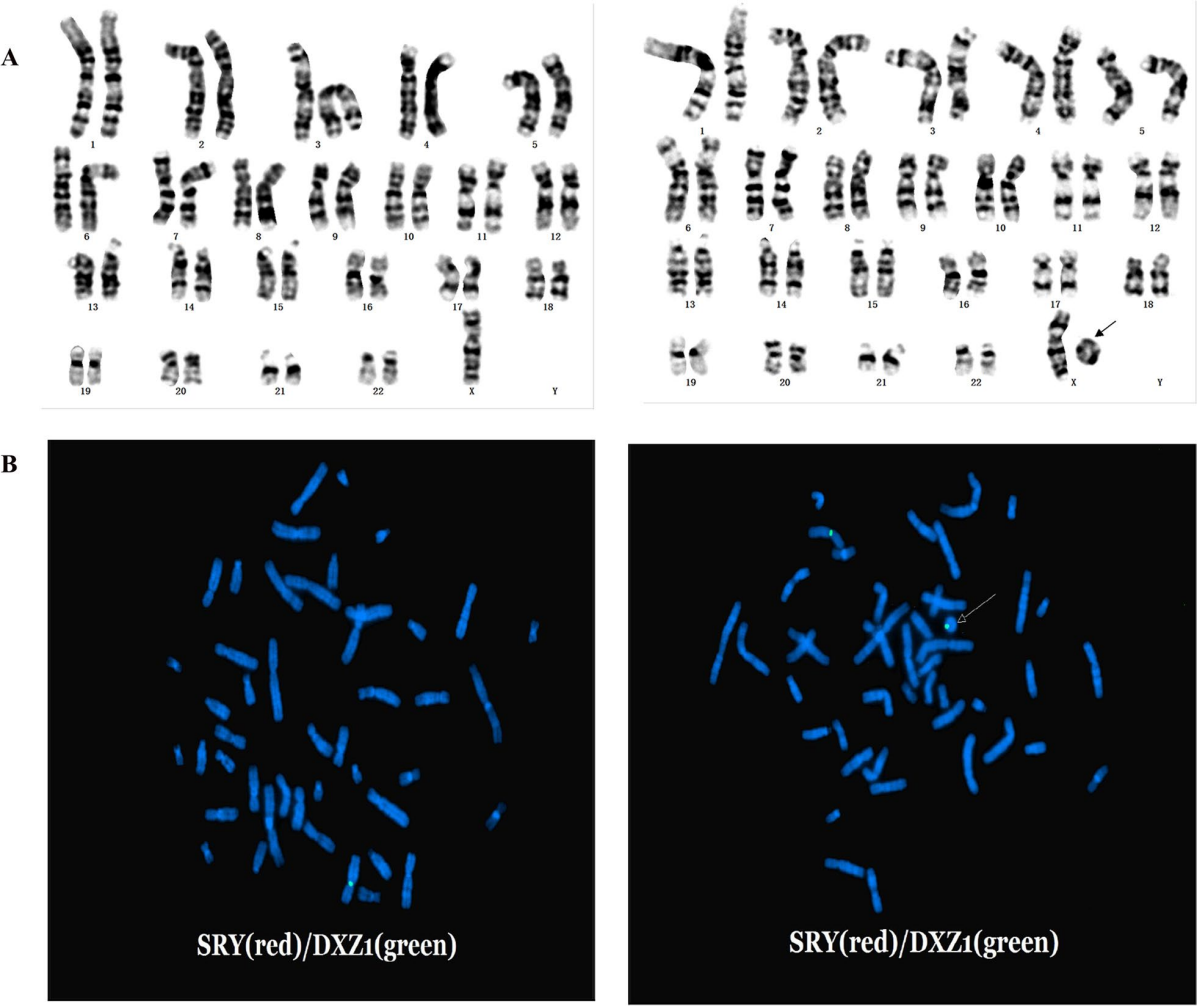
Karyotype analysis. A, High resolution G-band karyotype (550–850 bands) of a cell showing 46 chromosomes, with an absent chromosome X (Left) and a small ring chromosome (Right). B, FISH with a specific centromeric X chromosome probe (*DXZ1)* showing two signals; one in the normal chromosome X and one in the small ring chromosome

In this case, the level of FSH and LH measured was 82.9 and 17.86 mIU/ml, respectively (Table 1). Both FSH and LH were measured at higher levels than normal (3.82–10.54 mIU/ml for FSH and 1.85–12.19 mIU/ml for LH). Karyotyping revealed mosaicism for a ring X chromosome in two types of cells, with karyotypes 45,X and 46,X,r (X) (Fig. 2). The proportion of cells with 46,X,r(X) was 30%. The origin of the ring chromosome was next determined by FISH with a probe for the peri centromeric region of the X chromosome. The degree of mosaicism was further confirmed by scoring FISH signals from metaphase and interphase cells (Fig. 2).

**Fig. 3:**
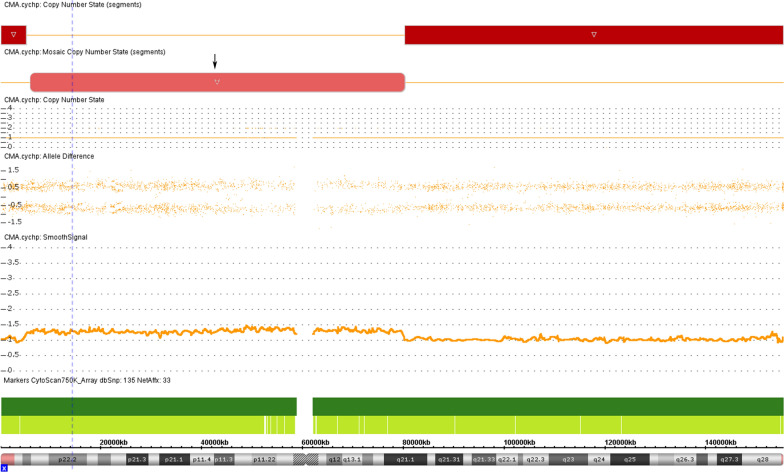
Chromosome microarray analysis shows loss of Mosaic arr[GRCh37] Xp22.33p22.32(168552_5051774) × 1, Xq21.1q28(80226215_155233098) × 1, Xp22.32q21.1(5905382_80163627) × 1–2. The pink section represents the lost Mosaic. The ring X chromosome was about 74 Mb in size. The genomic location was chrX:5905382–80163627

According to ISCN 2020, the microarray karyotype could be written as arr[GRCh37] Xp22.33p22.32(168552_5051774) × 1, Xq21.1q28(80226215_155233098) × 1, Xp22.32q21.1(5905382_80163627) × 1–2. Cytogenetic microarray analysis revealed the exact locations of the breakage points on the arms of the X chromosome [45,X/46,X,r(X)(p22.32q21.1)] (Fig. 3). The length of the r(X) chromosome was found to be 74 Mb (Xp22.32 –Xq21.1), harboring 363 OMIM genes. The degree of mosaicism was determined from the cytogenetic microarray experiment by comparing the deviation in the probe intensity signals from baseline (Fig. 3).

## Discussion

TS is a chromosomal disorder in females. Classic TS patients have the karyotype 45,X. Some other TS patients can present with mosaicism or have a structurally rearranged X karyotype [3]. Patients who carry a structurally abnormal X chromosome are a unique group, and they have provided opportunities to evaluate genotype/phenotype correlations in relation to X chromosome content and inactivation. Mosaicism karyotypes with structurally abnormal ring chromosomes are even rarer, accounting for only 5% of TS patients [2].

We here describe a 10 year old girl who had short stature and ovarian dysplasia. She was found to have a de novo mosaic 45,X/46,X, + r (p22.32q21.1) by cytogenetic analysis. The observed deletion of p22.32p22.33 [4.8 Mb] and Xq21.1-Xq28 was approximately 75 Mb in size, and harbored 605 genes. We found several similar cases associated with our case where a ring chromosome has been described previously. For example, the cases Xpter-Xp22.12 [21 MB] and Xq21.33-Xq28 [62 Mb] had more severe symptoms than our case, and these case’s deletion fragment contained 789 genes compared to our case, which only contained 605 [6]. This difference in symptom severity could be explained by the amount of X deletion and the number of genes contained in the deleted region. It was also possible that the difference in severity between the cases was due to differences in the degree of mosaicism for two cell lines 45, X and 46, Xr (X) (70:30 in ou case compared to 50:50 in the more sever case). However, it is difficult to ascertain the impact of mosaicism on clinical phenotype as level of mosaicism has been reported to go down with age due to the unstable nature of r (X) chromosomes.

Furthermore, we compared Xp deletions and clinical presentations in our case to those of previously reported cases that showed no evidence of ovarian dysgenesis with an Xp terminal deletion, Here, we report a rare case of TS with an X ring mosaicism karyotype of 45,X and 46,X, r(X)(p22.32q21.1). This case had an atypical phenotype. For example, her intellectual development did not lag behind her peers, and the patient’s breast development was Tanner stage 2. Other symptoms are described in Table 1. A case similar to ours (Xpter-Xp22.12 and Xpter-Xp22.31, respectively) had been reported in (Xp22.33-Xp22.12) without any sign of ovarian dysgenesis [7–9]. Therefore, we considered ovarian dysgenesis in our case might have been due to Xq deletion rather than Xp deletion.

Previous studies have found that ring X chromosomes, which are active, as defined by deficient *XIST* transcription, are present in females who have mental retardation and multiple congenital abnormalities [10, 11]. Small ring (X) chromosomes lacking the *XIST* gene have been associated with a severe phenotype that includes mental retardation, facial dysmorphism, and congenital abnormalities [10, 12]. It has been hypothesized that the loss of *XIST* results in functional disomy for the sequences contained in the ring. However, in our case, *XIST* was intact with the ring chromosome negating the role of *XIST* inactivation in the clinical phenotype of patients. Therefore, we believe that the results of ring X formation was most likely inactivation in our case.

Moreover, the loss of different genetic material leads to different clinical phenotypes in Turner syndrome, and the chromosomal karyotype and clinical phenotype are dependent. Short stature is the most common characteristic, and is caused by the perturbations of the *SHOX* gene on Xp22.3 [7, 8, 13]. The key region determining the development of female gonads is in Xq21-Xq27, and the disruption of these regions will lead to female ovarian dysplasia [14, 15]. Our case was consistent with the phenotype reported in previous studies. The clinical phenotype of our case was less severe than that of a classic 45X karyotype, which may be related to mosaicism of the ring X chromosome, but this is still as uncertain.

In summary, we describe the phenotype in terms of clinical symptoms, ultrasound diagnosis, and cytogenetics, of a girl with a rare genotype and TS. We discussed the similarities and differences between our case and other previously reported cases with similar genotypes. We used a combination of techniques and analysis of information from public gene databases (Decipher/Clingen/UCSC/Pubmed) to provide additional evidence to clarify the genotype-phenotypic associations of TS, which can facilitate further genetic counseling and enrich database information on TS.

## Data Availability

The datasets used and/or analyzed in the current study are available from the corresponding author upon reasonable request.
